# The gene regulatory network for breast cancer: integrated regulatory landscape of cancer hallmarks

**DOI:** 10.3389/fgene.2014.00015

**Published:** 2014-02-03

**Authors:** Frank Emmert-Streib, Ricardo de Matos Simoes, Paul Mullan, Benjamin Haibe-Kains, Matthias Dehmer

**Affiliations:** ^1^Computational Biology and Machine Learning Laboratory, Faculty of Medicine, Health and Life Sciences, Center for Cancer Research and Cell Biology, School of Medicine, Dentistry and Biomedical Sciences, Queen's University BelfastBelfast, UK; ^2^Faculty of Medicine, Health and Life Sciences, Center for Cancer Research and Cell Biology, School of Medicine, Dentistry and Biomedical Sciences, Queen's University BelfastBelfast, UK; ^3^Bioinformatics and Computational Genomics Laboratory, Princess Margaret Cancer Centre, University Health NetworkToronto, Ontario, Canada; ^4^Institute for Bioinformatics and Translational Research, UMIT, Eduard Wallnoefer Zentrum 1Hall in Tyrol, Austria

**Keywords:** breast cancer, gene regulatory network, BC3Net, GPEA, statistical inference, computational genomics

## Abstract

In this study, we infer the breast cancer gene regulatory network from gene expression data. This network is obtained from the application of the BC3Net inference algorithm to a large-scale gene expression data set consisting of 351 patient samples. In order to elucidate the functional relevance of the inferred network, we are performing a Gene Ontology (GO) analysis for its structural components. Our analysis reveals that most significant GO-terms we find for the breast cancer network represent functional modules of biological processes that are described by known cancer hallmarks, including translation, immune response, cell cycle, organelle fission, mitosis, cell adhesion, RNA processing, RNA splicing and response to wounding. Furthermore, by using a curated list of census cancer genes, we find an enrichment in these functional modules. Finally, we study cooperative effects of chromosomes based on information of interacting genes in the beast cancer network. We find that chromosome 21 is most coactive with other chromosomes. To our knowledge this is the first study investigating the genome-scale breast cancer network.

## 1. Introduction

Breast cancer is among the most common cancers with worldwide more than 1,300,000 cases each year (Cancer and Atlas, 2012). Among these cases, ductal carcinoma, a particular subtype of breast cancer, represents up to 25% of all newly diagnosed patients in the United States (Wickerham and Julian, 2013). In general, breast cancer is derived from epithelial cells that develop neoplasia in breast tissue. In the malignant case these cells are invasive and can become metastatic. It is known that the major cancer hallmarks, common to all mammalian cancer forms, are related to cell differentiation, proliferation and cell apoptosis processes that are associated to the deregulation of the cell cycle and impairment of DNA repair processes (Hanahan and Weinberg, 2000, 2011a). This makes cancer a disease of pathways (Hanahan and Weinberg, 2000). Unfortunately, the underlying molecular interactions of these processes are to-date not well understood and the corresponding network of the mechanistic interplay and physical interactions between individual genes, their products, proteins and metabolites is underexplored. A reason for this lack of knowledge is due to the fact that most pathways do not have a chain-like structure, but are complex connected to regulate particular cellular processes and responses. This makes cancer a complex disease, difficult to study as it can not be traced back to an individual component, e.g., a protein in the network. For this reason, cancer genes need to be understood as being part of a complex network and the malfunction of a process may be caused by inadequate interactions (Kitano, 2004). Although major efforts have been made to identify important interaction partners in various cancer types (Basso et al., 2005; Madhamshettiwar et al., 2012; de Matos Simoes et al., 2013a), the actual interaction structure of genome-scale networks is far from being known.

In general, genes involved in the development and progression of cancer represent a broad class of proteins such as transcription factors, chromatin remodelers, growth factors (e.g., EGFR), growth factor receptors (e.g., HER2/neu), signal transducers, regulators of apoptosis and DNA repair genes (Croce, 2008). The individual key players of cancer progression are classified as (A) oncogenes, (B) tumor suppressor genes and (C) genomic stability genes (Vogelstein and Kinzler, 2004). These genes are playing a key role in the regulation of the cell-cycle, proliferation and cell differentiation, and in the regulation of apoptosis (Croce, 2008). Specifically, oncogenes accumulate particular mutations that lead to a constitutive structural active form of the protein. In contrast, specific mutations in tumor suppressor genes (e.g., APC) lead to an inactivation or decreased activity of the protein. Stability genes include proteins involved in DNA repair, mitotic recombination and chromosome segregation (e.g., BRCA1). It is important to note that cancer genes are mostly identified by their genetic alterations such as germline or somatic mutations, which are sequenced from tumor tissues (Sjoblom et al., 2006). Specifically, mutations in cancer genes can be somatic or germlime genomic single nucleotide substitutions, deletions, insertions (Futreal et al., 2004) or mutations of larger DNA segments that are amplified, translocated, deleted or inserted (Mitelman, 2000).

Due to complex nature of breast cancer, we are pursuing in this paper a systems approach (Alon, 2006; Hernandez-Lemus, 2013) based on *gene regulatory networks*. Specifically, a gene regulatory network (GRN) is a description of the complex molecular interactions between individual genes and their products (Hecker et al., 2009; Emmert-Streib et al., 2012a,b). Statistically, gene regulatory networks are inferred from large-scale gene expression data from a variety of cancer tissue samples and many contemporary inference methods are based on estimates of mutual information (Li, 1990; Steuer et al., 2002; Meyer et al., 2008; Emmert-Streib et al., 2012a). Generally, biological networks have been analyzed structurally by using entropy-based network measures and other quantities which characterize the underlying graph topology uniquely (Mueller et al., 2011; Dehmer et al., 2012).

The major goal of this paper is to use the BC3NET method (de Matos Simoes and Emmert-Streib, 2012) for inferring a breast cancer gene regulatory network. Specifically, we are using 351 breast cancer samples from the Expression Project for Oncology (expO) (http://www.intgen.org/expo/) microarray database maintained by the International Genomics Consortium (IGC). For this breast cancer gene regulatory network, we perform a systems analysis with respect to its functional and structural features. Furthermore, we study the role of known general cancer genes and specific breast cancer genes in the breast cancer network. Finally, we investigate cooperative effects between chromosomes by relating interactions back to their chromosomal positions.

This paper is organized as follows: In the next section, we describe all methods and data we are using for our analysis. In the results section, we present our findings and in the section “Discussion” we interpret our results. The paper finishes with the section “Conclusions” with a summary.

### 1.1. Author summary

**What types of biological networks have been inferred in the paper?** We use gene expression data from breast cancer samples and infer a gene regulatory network (GRN).

**How was the quality/utility of the inferred networks assessed?** We assess the biological validity of the inferred GRN by using the Gene Ontology database.

**How were these networks validated?** The GRN is analyzed computationally and statistical hypotheses testing is employed to test various hypotheses about the network structure and the biological function of the investigated GRN of breast cancer.

## 2. Methods

### 2.1. Breast cancer gene expression data

For our study, we use 351 breast cancer tissue samples from the Expression Project for Oncology (expO) (http://www.intgen.org/expo/) microarray database maintained by the International Genomics Consortium (IGC). ExpO provides breast cancer samples from histologically determined tumors of various types, see Table 1 for an overview, whereas the majority of samples (over 80%) is from ductual carcinoma across various grades (1, 2, and 3) and stages (1, 2A, 2B, 3A, 3B, 3C, and 4). The majority of patients are in the age group between 40 and 70 (238/351 patients). Most of the patient samples have a caucasian ethnic background (314/351 samples). A total of 136/351 patients were without family history of cancer and 213/351 were with a family history of cancer (two samples have unknown family history of cancer). 346 samples are from female and 5 from male gender. The 351 breast cancer Affymetrix *hgu133plus2* microarray samples in *CEL* format were obtained from the GEO NCBI repository (accession number *GSE2109*) (Edgar et al., 2002).

**Table 1:** **Overview of histological annotations of the 351 samples of the expO breast cancer gene expression compendium**.

**Histology description**	**Samples**
Ductal carcinoma	270
Lobular carcinoma	39
Invasive ductal carcinoma	7
Mucinous carcinoma	6
Ductal and lobular carcinoma	4
Metaplastic carcinoma	3
Metaplastic squamous carcinoma	3
Cribiform carcinoma	2
Inflammatory carcinoma	2
Intraductal carcinoma	2
Intraductal papillary carcinoma	2
Medullary carcinoma, NOS	2
Adenoid cystic carcinoma	1
Colloid adenocarcinoma	1
Ductal carcinoma *in situ* (DCIS) with focal comedo carcinoma	1
Intracystic carcinoma	1
Invasive ductal carcinoma, mucinous type	1
Lobular carcinoma *in situ*	1
Papillary carcinoma	1
Papillary carcinoma (predominantly micropapillary pattern)	1
Unknown	1

#### 2.1.1. Preprocessing and normalization

We normalize the microarray samples for the selected tissue types using RMA and quantile normalization (Irizarry et al., 2003) using *log*_2_ expression intensities for each probe set. Because a gene can be represented by more than one probe sets, we use the median expression value as summary statistic for different probe sets. Entrez gene ID to Affymetrix probe set annotation is obtained from the *“hgu133plus2.db”* R package. If a probe sets is unmapped, we exclude it from our analysis. After these preprocessing steps, we have 19,738 genes and 351 samples we use for our analysis.

### 2.2. BC3NET

In order to infer the gene regulatory network for the gene expression data from breast cancer, we use the BC3NET algorithm (de Matos Simoes and Emmert-Streib, 2012) to infer a mutual information based gene regulatory network. In the following, we denote this network briefly as *G*_BC3Net_. The gene regulatory network *G*_BC3Net_ is inferred from a bootstrap ensemble generated from a single gene expression dataset *D*. In the first step of the procedure mutual information values among all gene pairs are estimated using the Pearson estimator. In the second step, for each gene at most one gene is selected for each of the *p* genes in a given dataset to contribute *at most* one edge to the inferred network. In overall *p* different null hypotheses for mutual independency are tested. In the third step we control the type one error by applying a Bonferroni multiple testing procedure. This results in a network *G^b^_k_* that is inferred for each *k* of 100 Bootstrap datasets. For each generated dataset in the ensemble, *D^b^_k_*, a network, *G^b^_k_*, is inferred using C3NET (Altay and Emmert-Streib, 2010). From {*G^b^_k_*}*^B^*_*k* = 1_ an aggregated network *G^b^_w_* is obtained whose edges are used as test statistics to obtain the final network *G*. The test statistic for each edge is used for a binomial test to test for significance of the connection between gene pairs BC3NET. If a connection between a gene pair is statistically significant (α ≤ 0.05) they are connected by and edge, otherwise there is no connection.

### 2.3. Cancer gene census

We use the complete list of the Cancer Gene Census (CGC) (Futreal et al., 2004) (Version 2011−03−22, *Table*_1_*full*_2011−03−22) (http://www.sanger.ac.uk/genetics/CGP/Census/). The CGC list comprises a total of 457 cancer genes. From these 457 genes, 435 are present in the expO breast cancer gene expression data set. The manually curated CGC list contains genes reported in the literature having cancer associated somatic or germline non-synonymous substitutions, insertions and deletions in coding regions or splice sites and genes affected by chromosomal rearrangements or copy number variations (Futreal et al., 2004).

### 2.4. Degree distribution

In order to assess the global connectivity of the inferred breast cancer network we estimate its degree distribution for a power-law distribution (Barabási and Albert, 1999; Newman, 2005). The degree distribution of a power-law follows

(1) $$P(k)\sim k^{-\alpha }$$

whereas α is the exponent of the power-law distribution.

### 2.5. GPEA: *g*ene *p*air *e*nrichment *a*nalysis

In order to test the enrichment of Gene Ontology (GO)-terms in the inferred breast cancer network, we are applying a hypergeometric test for edges (gene pairs), instead of genes. For this reason, this analysis is called *g*ene *p*air *e*nrichment *a*nalysis (GPEA) (de Matos Simoes and Emmert-Streib, 2012; de Matos Simoes et al., 2013b).

For *p* genes there is a total of *N* = *p*(*p* − 1)/2 different gene pairs. If there are *p*_GO_ genes for a particular GO-term then the total number of gene pairs for this GO-term is *m*_GO_ = *p*_GO_(*p*_GO_ − 1)/2. Suppose the inferred breast cancer network contains *n* edges, of which *k* are edges are among genes from the given GO-term. Then a *p*-value for the enrichment of this GO-term can be calculated from a hypergeometric distribution by

(2) $$\begin{array}{l} p(k|\text{GO}-\text{term})=\sum_{i=k}^{m_{\text{GO}}}P(X=i|\text{GO}-\text{term})\\ \;=\sum_{i=k}^{m_{\text{GO}}}\frac{\left(\begin{array}{c} m_{\text{GO}}\\ i \end{array}\right)\left(\begin{array}{c} N-m_{\text{GO}}\\ n-i \end{array}\right)}{\left(\begin{array}{c} N\\ n \end{array}\right)} \end{array}$$

Here the *p*-value gives an estimate for the probability to observe *k* or more edges between genes from the given GO-term. We access the GO annotation for entrez identifiers from the Bioconductor (Gentleman et al., 2004) annotation packages *org.Hs.eg.db* (v2.9.0) and *GO.db* (v2.9.0).

## 3. Results

### 3.1. Breast cancer gene regulatory network

Using the expO data set and the BC3NET algorithm, we infer a breast cancer gene regulatory network (GRN). In the following, we denote this network briefly as *G*_BC3Net_. This regulatory network consists of 19,738 genes and contains 180,171 interactions (edges) among these genes. With the exception of 15 genes the overall network is connected, i.e., we can always find a path that connects a pair of genes with each other. Technically, this means that the giant connected component (GCC) (Dorogovtesev and Mendes, 2003) of our breast cancer GRN has a size of 19,723 genes. For this network, we find an average shortest path length of 4.11 and its edge density is ϵ = 9.2 · 10^−4^. Here we measure a shortest path by means of the Dijkstra distance (Dijkstra, 1959). Furthermore, we find the degree distribution of the network to follow a power law distribution with an exponent of α = 3.48. This indicates that the resulting network is a *scale-free* network (Barabási and Albert, 1999) as found for many different types of biological networks (Bornholdt and Schuster, 2003; van Noort et al., 2004; Albert, 2005; Basso et al., 2005).

### 3.2. Functional analysis of biological processes using GPEA

In order to evaluate the inferred breast cancer GRN biologically, we use the GO database (Ashburner et al., 2000). Specifically, we evaluate our network based on functional knowledge about genes that are involved in similar biological processes. We are interested to identify which functional modules are most prominently represented in our inferred breast cancer network under the assumption that functionally related genes are likely to interact with each other. Furthermore, we want to identify which known cancer genes are represented (enriched) in those functional modules. This will shed light on the role and importance of cancer genes in the breast cancer network.

We conduct this functional analysis of the breast cancer network by using the GPEA (gene pair enrichment analysis; see “Methods” section) method. The results of this analysis are shown in Table 2. Briefly, a GPEA analysis identifies GO-terms with an enriched number of interactions among genes from the same GO category. We correct for multiple testing using a Bonferroni correction for a significance level of α = 0.05. In order to assess the role of census genes for the individual GO-terms we counted the number of census genes present in each GO-term. For the analysis, we consider a total of 7989 GO-terms from the category Biological Process, with a term size larger than 2 and less than 1000 genes. In total, we find 632 enriched GO-terms (12.64%). The 50 most significant terms of the GPEA analysis are shown in Table 2. As one can see, the significant terms describe a variety of biological processes such as mitotic cell cycle (1031 edges), cell cycle phase (1142 edges), mRNA translation such as translational elongation (218 edges), termination (191 edges) and initiation (226 edges), protein targeting to ER (196 edges), viral transcription (193 edges), protein complex disassembly (197 edges), regulation of immune system process (827 edges), innate immune response (368 edges), cell adhesion (867 edges) and type I interferon-mediated signaling pathway (71 edges).

**Table 2:** **GPEA analysis of the breast cancer gene regulatory network for GO biological processes**.

**GOID**	**GO term**	**Size**	**Edges**	***p*-value**	**CG**
GO:0000278	Mitotic cell cycle	776	1031	2.3e-260	54/+
GO:0006414	Translational elongation	108	218	6.7e-259	1
GO:0022403	Cell cycle phase	853	1142	1.1e-257	60/+
GO:0006415	Translational termination	91	191	3.5e-244	1
GO:0006614	SRP-dependent cotranslational protein targeting to membrane	105	195	9.1e-227	2
GO:0045047	Protein targeting to ER	107	196	3.6e-225	2
GO:0072599	Establishment of protein localization to endoplasmic reticulum	108	196	1.4e-223	2
GO:0006613	Cotranslational protein targeting to membrane	107	195	1.4e-223	2
GO:0000184	Nuclear-transcribed mRNA catabolic process, non-sense-mediated decay	118	198	1.1e-211	2
GO:0070972	Protein localization to endoplasmic reticulum	121	197	6.2e-206	2
GO:0006413	Translational initiation	153	226	1.0e-204	4
GO:0000087	M phase of mitotic cell cycle	374	415	2.3e-182	20/+
GO:0000280	Nuclear division	363	391	1.0e-171	20/+
GO:0007067	Mitosis	363	391	1.0e-171	20/+
GO:0000279	M phase	537	573	1.7e-171	33/+
GO:0006612	Protein targeting to membrane	154	200	1.1e-169	4
GO:0000956	Nuclear-transcribed mRNA catabolic process	171	212	1.0e-166	7
GO:0048285	Organelle fission	388	409	1.6e-166	20/+
GO:0019080	Viral genome expression	152	193	6.4e-163	10/+
GO:0019083	Viral transcription	152	193	6.4e-163	10/+
GO:0043624	Cellular protein complex disassembly	157	196	1.8e-161	2
GO:0043241	Protein complex disassembly	162	197	1.3e-157	2
GO:0006402	mRNA catabolic process	183	213	4.3e-156	7
GO:0032984	Macromolecular complex disassembly	183	201	1.8e-142	7
GO:0006401	RNA catabolic process	210	218	1.1e-137	7
GO:0072594	Establishment of protein localization to organelle	212	213	6.1e-131	4
GO:0019058	Viral infectious cycle	228	218	1.4e-123	14/+
GO:0051301	Cell division	480	419	1.3e-112	35/+
GO:0016071	mRNA metabolic process	614	544	1.3e-107	21/+
GO:0006412	Translation	469	376	2.3e-93	16/+
GO:0002682	Regulation of immune system process	893	827	7.1e-91	83/+
GO:0022411	Cellular component disassembly	295	229	8.5e-90	12/+
GO:0001775	Cell activation	763	663	6.4e-88	74/+
GO:0046649	Lymphocyte activation	471	367	2.8e-87	61/+
GO:0045321	Leukocyte activation	556	439	2.3e-84	63/+
GO:0050776	Regulation of immune response	564	435	8.7e-79	43/+
GO:0022415	Viral reproductive process	547	416	3.0e-77	44/+
GO:0007155	Cell adhesion	963	867	1.4e-74	41/+
GO:0022610	Biological adhesion	965	869	2.1e-74	41/+
GO:0060337	Type I interferon-mediated signaling pathway	73	71	8.5e-73	5/+
GO:0071357	Cellular response to type I interferon	73	71	8.5e-73	5/+
GO:0034340	Response to type I interferon	74	71	5.7e-72	5/+
GO:0016032	Viral reproduction	701	546	1.6e-68	46/+
GO:0044764	Multi-organism cellular process	703	547	4.0e-68	46/+
GO:0006396	RNA processing	656	488	1.4e-63	18
GO:0010564	Regulation of cell cycle process	440	295	2.9e-62	45/+
GO:0002684	Positive regulation of immune system process	558	387	1.9e-59	41/+
GO:0006259	DNA metabolic process	880	707	1.2e-56	75/+
GO:0051249	Regulation of lymphocyte activation	296	183	1.5e-56	34/+
GO:0045087	Innate immune response	544	368	1.8e-56	25/+

*Shown are the 50 most significant terms (p-values are Bonferroni adjusted). CG is the number of cancer genes present in each term and the “+” indicates the significant enrichment of a GO-term with cancer genes. The total number of census genes in these 50 GO-terms is 256*.

Interestingly, the significant biological processes shown in Table 2 are known to be most affected in breast cancer and many are recognized as hallmarks of cancer. For example, increased translational initiation through elevated expression of key genes such as pS6, p4E-BP1, eEF2K, and decreased pdcd4 are associated with poor prognosis in hormone receptor-positive breast cancer, highlighting the role of translational control in breast cancer pathogenesis (Meric-Bernstam et al., 2012).

Also, inflammation has been cited as one of the major hallmarks of cancer and immune infiltration of tumors (principally by the innate immune system) has been shown to be a key component in both the initiation, progression, survival rates and chemotherapy responses of multiple cancer types including breast cancer (DeNardo et al., 2011; Hanahan and Weinberg, 2011b). An emerging hallmark of cancer is its ability to evade the host immune system by paralyzing immune cells such as CTLs and NK cells through secretion of TGF? or other immunosuppressive mediators (Shields et al., 2010). Aneuploidy and Chromsomal Instability (CIN) are also well known hallmarks of cancer which highlight the dysregulation of mitotic control and chromosome segregation in many cancer types. Many mitotic regulators are known to be overexpressed principally by transcriptional upregulation (Aurora kinases, PLKs) or less frequently mutated (Bub1, Bub1R, Mps1) resulting in impaired mitotic checkpoints (reviewed in Kops et al., 2005).

#### 3.2.1. Cancer census genes and cell cycle

To study the relationship of the identified functional modules and known cancer genes, we utilize the manually curated cancer gene census (CGC) list (Futreal et al., 2004) consisting in total of 435 cancer genes. For each GO-term, we count the number of present cancer genes (last column in Table 2) and perform a hypergeometric test to determine the enrichment of cancer genes. From the 50 tests for each GO-term in Table 2, we find 32 to be enriched with cancer genes; after a Bonferroni correction for a significance level of α = 0.05. These GO-terms are highlighted by a “+” in the last column in Table 2. Furthermore, the 50 most significant GO-terms comprise in total 4743 genes, of which 238 are cancer genes (54.71% = 256/435). Also this gene set comprising all 50 GO-terms is significantly enriched with cancer genes.

**Figure 1:**
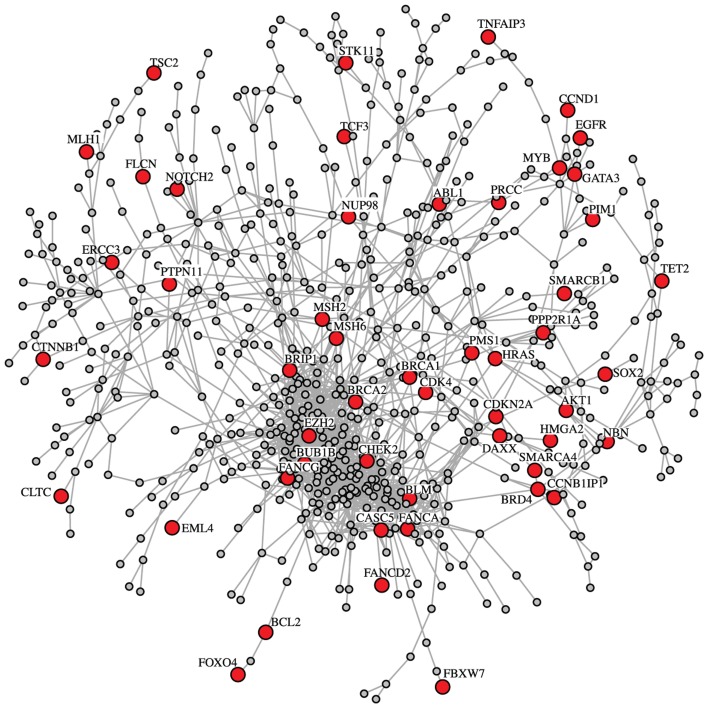
**Subnetwork of the breast cancer GRN for the biological process cell cycle (GO:0007049)**. This subnetwork includes 51 census cancer genes, e.g., *BRCA1* and *BRCA2*. The census cancer genes are highlighted in red. Overall, the shown network consists of 728 genes and 1671 interactions.

In Figure 1, we show a subnetwork of our breast cancer GRN that includes only genes belonging to the biological process term *cell cycle (GO:0007049)*. This network component contains a total of 345 interactions among 728 genes of which 51 genes are known cancer genes (Futreal et al., 2004). Among these 51 cancer genes, we find *BRCA1* and *BRCA2* that are multifunctional proteins playing a major role in DNA repair processes.

**Figure 2:**
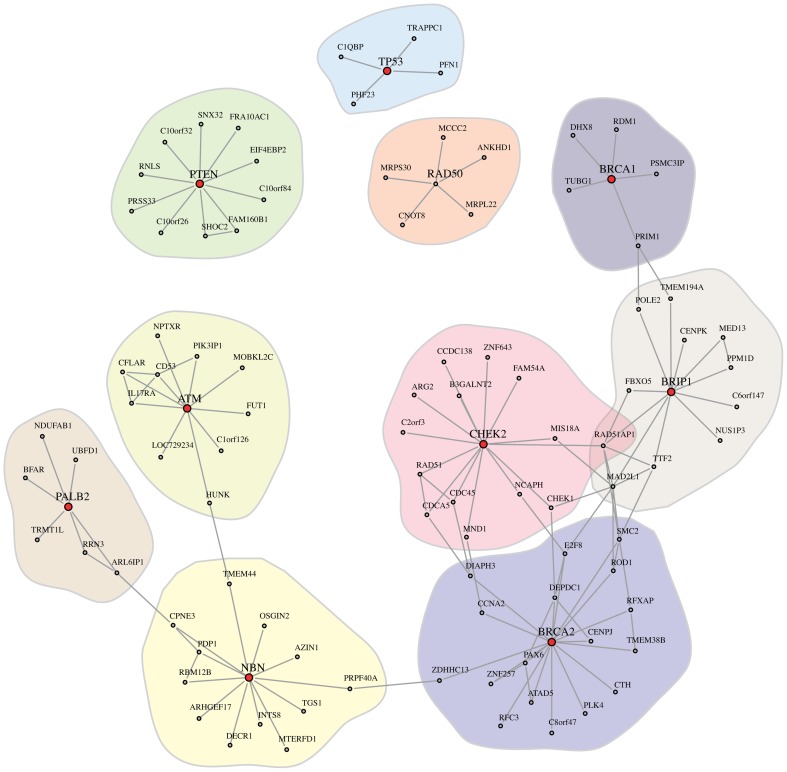
**Shown is the 1st degree neighbors subnetwork, G^1st^, of the cancer genes *BRCA1*, *BRCA2*, *TP53*, *PTEN*, *CHEK2*, *ATM*, *NBN* (NBS1), *RAD50*, *BRIP1*, and *PALB2* (highlighted in red), extracted from G_BC3Net_**. Note that we also included RAD50, despite the fact that RAD50 is not present in cancer census gene list.

Interestingly, our breast cancer GRN shows proximities of some well characterized interactions. For example, Figure 1 shows a close association of BRCA2, MSH2, and MSH6. These proteins are known to interact in the BRCA1 Associated Surveillance Complex (BASC), a large multicomponent DNA damage sensing complex containing proteins with roles in recognition of abnormal DNA structures or damaged DNA, suggesting that BASC may serve as a sensor for DNA damage (Wang et al., 2000). Additionally Figure 1 shows a close association between FANCA and BLM. Both proteins again have been shown to interact in a multiprotein complex and participate in genomic maintenance (Meetei et al., 2003). Within the 1st level neighbors shown in Figure 2 there also appear to be interesting associations. For example, p53 is proposed to closely associate with C1QBP a protein modulated EGF-induced cancer cell chemotaxis and metastasis in Severe Combined Immunodeficiency (SCID) mouse models, suggesting that p53 loss of function could result in C1QBP-mediated metastatic events (Zhang et al., 2013). Conversely, p53 also showed close association with PFN1 a protein shown to have antiproliferative function in MDA-MB-231 cells (Zou et al., 2010).

### 3.3. Local landscape of breast cancer genes

Next, we investigate 10 well-known genes that are frequently observed in inherited breast cancer. Specifically, germline mutations in *BRCA1*, *BRCA2*, *TP53*, *PTEN*, *CHEK2*, *ATM*, *NBN* (also denoted by NBS1), *RAD50*, *BRIP1*, and *PALB2* are known to be associated with a high risk for breast cancer (Walsh and King, 2007). Interestingly, all of these genes, except RAD50, are also in the cancer census gene list (Futreal et al., 2004) and it is known that these genes are playing an important role in genomic integrity such as DNA repair pathways.

In order to study the local interaction landscape of these 10 breast cancer genes, we extract their 1st degree neighbors from our breast cancer network *G*_BC3Net_. Figure 2 shows the resulting subnetwork, which we denote by *G*^1st^. As one can see, we obtain one large network component in *G*^1st^ that includes seven cancer genes (*BRCA1*, *BRCA2*, *CHEK2*, *ATM*, *NBN* (NBS1), *BRIP1*, and *PALB2*) and three smaller components that contain only a single cancer gene (*TP53*, *PTEN*, and *RAD50*).

Overall, *G*^1st^ consists of 116 genes and 209 interactions. We would like to emphasize that despite the fact that in *G*^1st^ (Figure 2), e.g., TP53 is not connected to BRCA1, there is a path in our breast cancer network *G*_BC3Net_. The reason for this is that *G*^1st^ contains only the 1st degree neighbors of the 10 breast cancer genes in *G*_BC3Net_ in order to obtain a (small) subnetwork that can be visualized sensibly. Extracting the subnetwork from *G*_BC3Net_ that would connect all 10 cancer genes with each other along shortest paths consists of 107 genes and has an average shortest path length of 4.31. In Table 3 we show the length of the shortest paths that connect these 10 breast cancer genes.

**Table 3:** **Length of the shortest path between pairs of breast cancer genes (Bgene1 and Bgene2)**.

**Bgene1**	**Bgene2**	**Shortest path length**
CHEK2	BRIP1	2
BRCA1	BRIP1	3
BRCA2	CHEK2	3
BRCA2	NBN	3
BRCA2	BRIP1	3
ATM	NBN	3
NBN	PALB2	3
BRCA1	BRCA2	4
BRCA1	CHEK2	4
BRCA1	PALB2	4
BRCA2	PTEN	4
BRCA2	RAD50	4
BRCA2	PALB2	4
TP53	PALB2	4
PTEN	CHEK2	4
PTEN	NBN	4
PTEN	RAD50	4
CHEK2	ATM	4
CHEK2	NBN	4
CHEK2	RAD50	4
CHEK2	PALB2	4
NBN	BRIP1	4
RAD50	BRIP1	4
RAD50	PALB2	4
BRIP1	PALB2	4
BRCA1	PTEN	5
BRCA1	ATM	5
BRCA1	NBN	5
BRCA1	RAD50	5
BRCA2	TP53	5
BRCA2	ATM	5
TP53	PTEN	5
TP53	CHEK2	5
TP53	ATM	5
TP53	NBN	5
TP53	RAD50	5
TP53	BRIP1	5
PTEN	ATM	5
PTEN	BRIP1	5
PTEN	PALB2	5
ATM	RAD50	5
ATM	BRIP1	5
NBN	RAD50	5
BRCA1	TP53	6
ATM	PALB2	6

Biologically, the interconnectivity between the genes in *G*^1st^ shown in Figure 2 reflects the combined roles of these individual genes in DNA damage signalling and repair, which is a major feature of cancer predisposition in breast cancer. For example, both BRCA1 and BRCA2 play key roles in co-ordination of homologous recombination events following double strand break repair (DSBR) (for review see Powell et al., 2011). Whilst BRCA1 and BRCA2 are considered “high risk” the other five members of this network component are considered “moderate risk” with a two- to fourfold increased breast cancer risk relative to the general population (10%) (Hollestelle et al., 2010). BRIP1 (also known as FANCJ or BACH1) and PALB2 physically interact with BRCA1 and BRCA2 to orchestrate helicase unwinding of DNA and to promote RAD51-mediated strand invasion functions in DSBR. A common DNA damage network between the BRCA and Fanconi anaemia (FA) pathways has been proposed and three FA genes, FANCD1, FANCN, and FANCJ, are identical to the BRCA genes BRCA2, PALB2, and BRIP1 (reviewed in Wang, 2007). ATM acts as a sentinel kinase detecting and signalling DSBs through phosphorylation of a plethora of other proteins including NBS1 (to initiate end processing of DSB ends as part of the MRN complex) and CHEK2 to initiate the enforcement of cell cycle arrest. Whilst RAD50 and p53 constitute separate modes in this regulatory network, they are nevertheless integrated into DSBR signalling, notably the MRN end processing of DSBs and transcriptional regulation of cell cycle arrest, respectively. PTEN, a substrate of ATM with functions related in DNA damage repair signalling (Bassi et al., 2013), also has distinct tumor suppressor roles in modulation of PI3K activity and phosphatidyl inositol signalling.

### 3.4. Closeness and gene neighbor degree of census cancer genes

In this section we study the closeness between cancer genes in the breast cancer GRN. For the set of 435 cancer census genes (Futreal et al., 2004), present in the breast cancer GRN, we define census gene pairs that have a significant shorter shortest path length compared to all shortest path length of the entire network. The null distribution is obtained by the distribution of all shortest paths between all (19723^2^ − 19723)/2 = 194,488,503 gene pairs. For each of the (435^2^ − 435)/2 = 94,395 census gene pairs a *p*-value is estimated by the fraction of shortest path length from all gene pairs that are shorter as the observed shortest path of the census gene pair. We consider a multiple testing correction using the Benjamini and Hochberg procedure for α = 0.05.

As a result, we find 148 significant census gene pairs (0.15%), involving 188 (43.21%) cancer census genes, that have significant shorter shortest paths. Aside from this, we find a total of 51 network components of directly connected census genes. The largest network component of directly connected census genes is 21 and 11 genes with the remaining components with ≤ 8 genes.

### 3.5. Cooperation between chromosomes

Finally, we study the relation between the genetic context and the structural connectivity of our breast cancer network *G*_BC3Net_. We study this relation in the following way. First, we investigate the overall frequency of a gene pair being located on the same chromosome or located on different chromosomes. We find that, in average, 20.43% of the interactions in the breast cancer network connect genes that are located on the same chromosome. Hence, 79.57% of the interactions connect genes on different chromosomes. Interactions between genes on separate or the same chromosome can be seen as *trans-interactions* and *cis-interactions*, in analogy to the trans- and cis-regulation of genes (Cheung et al., 2010). However, we would like to emphasize that there is a crucial difference between both types of connections. For a “regulation”, the transcription of a gene is regulated by a cis- or trans-acting transcription factor, whereas an “interaction” means *any type* of biochemical binding, not limited to transcription regulation, but also including protein-protein interaction, phosphorylation, ubiquitination or others.

In our next analysis, we test if there are chromosome pairs that contain a statistically significant number of interactions. That means we calculate the number of interactions, e.g., between chromosome 2 and 21, denoted as *s*_2,21_, and apply a statistical hypothesis test to see if this number is larger than expected by chance, i.e., *s*_rand|2,21_. In order to obtain the a sampling distribution for the general null hypothesis

(3) $$H_{0}:s_{i,j}=s_{\text{rand}|i,j}$$

we randomize the gene labels in the breast cancer network *E* times. We would like to note that the indices *i* and *j* in *s*_rand|*i,j*_ indicate that the sampling distribution is different for each chromosome pair because it takes the varying size of the chromosomes into account. For each randomization, *e* ∈ *E*, we calculate the number of interactions *s^e^_i,j_* between each chromosome pair (*i*, *j* ∈ {1, 2, …, 22, *X*, *Y*}. From this, we estimate *p*-values by

(4) $$p_{i,j}=\frac{\sum_{e=1}^{E}I(s_{i,j}^{e}>s_{i,j})}{E}.$$

Here, *I*(), is the indicator function which gives a value of “1” if its argument is true and “0” otherwise. We would like to emphasize, first, that the way we estimate the *p*-values for each chromosome pair (*i,j*) uses its own, individual sampling distribution given by the values of {*s^e^_i,j_*}. Second, utilizing the connection structure of the inferred breast cancer network in combination with a gene label resampling conserves not only the total number of interactions among genes, but also the structural properties of the network. Furthermore, the uneven number of genes on the 24 chromosomes is considered by this procedure as well. In total, we perform 300 = (24^2^ − 24)/2 + 24 tests for chromosome pairs for the 24 chromosomes. In order to adjust for multiple testing, we apply a Benjamini and Hochberg (1995) correction controlling the FDR for a significance level of α = 0.05. This guarantees a false discovery rate of FDR ≤ α (Dudoit and van der Laan, 2007). For our non-parametric estimation of the *p*-values, we used *E* = 100,000.

**Figure 3:**
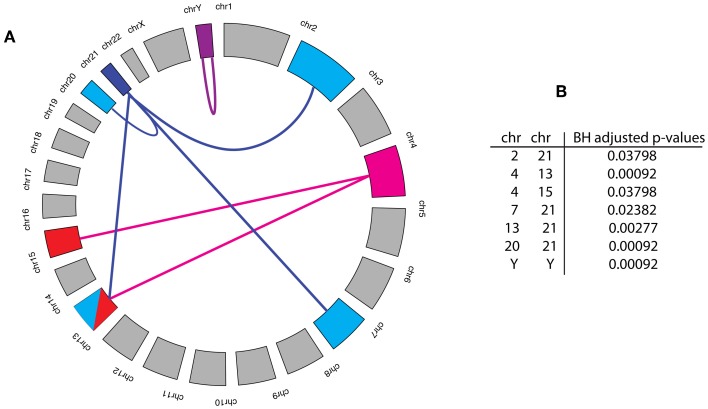
**(A)** Statistically significant cooperations between chromosome pairs are highlighted by a link. The Benjamini and Hochberg (BH) adjusted *p*-values for these links are shown in the Table **(B)**. The significance level for this analysis was α = 0.05, which guarantees that FDR ≤ α holds for the set of all significant links.

From our analysis, we find seven chromosome pairs that are statistically significant, shown in Figure 3B. Interestingly, 6 of the 7 significant pairs involve chromosome 4 and 21 and the remaining significant link represents a self-interaction on the Y chromosome. The results of our analysis shed light on the *cooperation* of genes as measured by the prevalence of significant interactions between chromosome pairs. From this perspective, visualized in Figure 3A, one sees that only a rather limited number of chromosomes contribute to this *cooperation* on the chromosome level.

In terms of intra-chromosomal gene co-regulation many low-penetrance breast cancer susceptibility loci are found to be located in non-protein-coding regions, suggesting their involvement in gene expression regulation. For example, Smits et al., have shown how the human MCS5A polymorphisms associated with breast cancer risk are located at both sides of a looped structure and functionally interact to downregulate transcriptional activity, a phenomenon which is conserved with rat Mcs5a [14]. In addition Akulenko and Helms have showed that out of 300 pairs of genes which showed co-methylation (but not nescessarily also co-repressed), 187 pairs were located on the same chromosome and were shown to be related to similar functional processes in the same pathways [15]. In fact they concluded that co-methylation “anti-correlated” with genomic distance [15]. Like most cancers, breast cancers are comprised not only of cancerous epithelia but also of numerous other contributory cell types which are involved in various stages of tumor initiation, progression and metastasis. There are instances where Loss of Heterozygosity (LOH) from genomic regions on the same chromosomes has been reported in breast cancer epithelial but furthermore LOH from adjacent regions could be detected in both breast cancer epithelia and breast cancer stromal cells from within the same tumors [16].

## 4. Discussion

In this study, we investigated a breast cancer gene regulatory network with respect to its structural and functional features. The network itself has been inferred from the application of BC3Net to a large-scale gene expression data set of breast cancer patients provided by the International Genomics Consortium (IGC) (expO data set).

From conducting a GPEA for *G*_BC3Net_, we found significant enrichment of GO-terms that represent biological processes for translation, immune response, cell cycle, organelle fission, mitosis, cell adhesion, RNA processing, RNA splicing and response to wounding. These biological processes are well described by cancer hallmark pathways (Hanahan and Weinberg, 2000, 2011b; Vogelstein and Kinzler, 2004; Berretta and Moscato, 2010) and known for playing central roles in differentiation, proliferation and immune system functional processes. In order to relate these processes to known cancer genes, we used the cancer census gene list (Futreal et al., 2004). Interestingly, our analysis revealed that 32 of the 50 most significant GO-terms are also enrichment with known cancer genes (see Table 2).

From studying a subnetwork of *G*_BC3Net_ limited to cell cycle genes, we found 51 known cancer genes. Among these 51 cancer genes the most prominent breast cancer genes *BRCA1*, *BRCA2*, and *Chk2* were present. *BRCA1* is multi-functional protein involved in DNA damage repair, cell cycle checkpoint activation, ubiquitination and in the regulation of gene expression (Zhu et al., 2011). *Chk2* is a tumor suppressor which functions as a protein kinase involved in DNA damage and cell-cycle arrest (Matsuoka et al., 1998). Although our GPEA analysis is restricted to the underlying biological knowledge gathered in the GO database, it provides a good overview of the most prominently represented biological processes present in the breast cancer network.

Currently, the analysis of genes involved in breast cancer is a very active field and resources have been established that provide a collective overview of such genes (Mosca et al., 2010). In our study, the manually curated census gene list (Futreal et al., 2004) has been used to gain functional insights of the involved biological processes. In previous studies, these cancer genes have been analyzed for a variety of cellular networks, e.g., protein interaction networks (Jonsson and Bates, 2006; Rambaldi et al., 2008) and manually curated signaling networks (Awan et al., 2007). For instance, a structural network analysis of the degree, betweenness and closeness of differentially expressed cancer genes, mapped on a protein interaction network, was performed by Hernandez et al. (2007). However, the main problem with such an approach is that the interactions in a protein network, e.g., measured by yeast-two-hybrid (Y2H), are outside of a disease context of the corresponding physiological state. On the other hand, one of the main advantages of gene regulatory networks is that they are measured in the *physiological context* under investigation. This should enable a more relevant analysis. A gene regulatory network also provides novel candidates for experimental investigations such as the hub genes that are highly enriched e.g., by membrane receptors (Table 4).

**Table 4:** **Census cancer genes ranked by degree in all-census-pair shortest path network**.

**Entrez ID**	**Deg. nodes**	**Symbol**	**Name**
81137	725	OR7E104P	Olfactory receptor, family 7, subfamily E, member 104 pseudogene
100132767	524		
2623	462	GATA1	GATA binding protein 1 (globin transcription factor 1)
387601	432	SLC22A25	Solute carrier family 22, member 25
353135	415	LCE1E	Late cornified envelope 1E
284187	384		
256646	334	C15orf55	Chromosome 15 open reading frame 55
219409	334	GSX1	GS homeobox 1
9362	326	CPNE6	Copine VI (neuronal)
283933	319	ZNF843	Zinc finger protein 843
9127	311	P2RX6	Purinergic receptor P2X, ligand-gated ion channel, 6
285877	303	POM121L12	POM121 transmembrane nucleoporin-like 12
9758	292	FRMPD4	FERM and PDZ domain containing 4
3166	292	HMX1	H6 family homeobox 1
60506	290	NYX	Nyctalopin
348808	288	NPHP3-AS1	NPHP3 antisense RNA 1
4584	287	MUC3A	Mucin 3A, cell surface associated
284805	283	C20orf203	Chromosome 20 open reading frame 203
163778	262	SPRR4	Small proline-rich protein 4
155	256	ADRB3	Adrenoceptor beta 3
286023	252	FLJ40288	Uncharacterized FLJ40288
64405	251	CDH22	Cadherin 22, type 2
1813	248	DRD2	Dopamine receptor D2
149647	245	FAM71A	Family with sequence similarity 71, member A
253868	238	C20orf166-AS1	C20orf166 antisense RNA 1

The impact of cancer progression driving gene mutations is explained by causing the malfunction of a protein, binding site alteration that causes a loss or constitutive deregulated function. Another major aspect of cancer progression is genome instability that causes larger mutation events, e.g., copy number variations, duplications, gene loss and translocation events of large genomic regions. Such processes can lead to the deregulation of genes and their related functions causing an amplification, downregulation or a complete shut-down of gene expression leading to a functional gain or loss. In order to relate the inferred interactions in our breast cancer network back to their genetic origin, we studied chromosomal effects. From this analysis, we found that in average 20.43% of the interactions in the breast network connect genes that are co-located on the same chromosome. Furthermore, we found only seven chromosome pairs, involving eight different chromosomes, that are more densely connected than expected by chance. Specifically, we observed the chromosomes 4, 13, and 21 to connect to two or more chromosomes and the chromosomes 2, 7, 15, 20, and Y to connect to exactly one chromosome. A putative explanation for this observation may be the amplification of the genes in a chromosome that would lead to a higher probability for interactions occurring within and between chromosomes.

On a more general note, one may wonder if the inferred gene regulatory network is “complete” or if parts of it, e.g., important driver gene(s) and their interactions, may be missing. Here it is crucial to realize that for our analysis, we used only *observational* data from cancer tissues. That means, the data were not generated in a controlled manner ensuring a sufficiently strong signal for all relevant biological components of the system. For this reason, it is possible that parts of the true breast cancer network were missed. Also, our network inference method BC3Net aims at inferring the strongest signal (network) within a given data set. However, from comparing information about our inferred gene regulatory network and independently conducted experiments one could get valuable information about such “missing parts” in order to come up with an experimental design that might fill-in these gaps. Hence, even information that is missing in our breast cancer network could be utilized for a future experimental design of follow-up studies.

## 5. Conclusions

Complex disorders like breast cancer require a systems level analysis. For this reason, network-based approaches provide a practical means to elucidate the biological function of processes from large-scale genomic data (Emmert-Streib and Dehmer, 2011). This also opens a venue for translation bioinformatics and personalized medicine, which depend crucially on the availability of robust, genome-scale analysis methods (Gonzalez-Angulo et al., 2010; Chan and Ginsburg, 2011; Chin et al., 2011).

### 5.1. Data sharing

We provide the gene expression data and the inferred breast cancer GRN from our analysis in the R-package BreastCancerGRN, available from CRAN.

### Conflict of interest statement

The authors declare that the research was conducted in the absence of any commercial or financial relationships that could be construed as a potential conflict of interest.
